# TLR4 rs4986790 and TLR9 rs5743836 Polymorphisms and Their Association With Clinical Outcomes of Leishmania infantum Infection in Tunisia

**DOI:** 10.7759/cureus.99686

**Published:** 2025-12-20

**Authors:** Najla Chargui, Nabil Mtiraoui, Raja Chaabane Banaoues, Hamouda Babba, Wahiba Sakly

**Affiliations:** 1 Clinical Research, Laboratory of Medical and Molecular Parasitology Mycology, Faculty of Pharmacy, University of Monastir, Monastir, TUN; 2 Laboratory of Human Genome and Multifactorial Diseases, Laboratory of Medical and Molecular Parasitology Mycology, Faculty of Pharmacy, University of Monastir, Monastir, TUN; 3 Clinical Biology, Laboratory of Medical and Molecular Parasitology Mycology, Faculty of Pharmacy, University of Monastir, Monastir, TUN

**Keywords:** cutaneous leishmaniasis, genetic association, leishmania infantum, polymorphism, snp, tlr4, tlr9, visceral leishmaniasis

## Abstract

Background: The clinical presentation of *Leishmania infantum* infection ranges from self-resolving cutaneous leishmaniasis (CL) to visceral leishmaniasis (VL). Host genetic factors, particularly polymorphisms in Toll-like receptor (*TLR*) genes, are suspected to influence disease variability and outcome. This study aimed to investigate the potential association between two specific single-nucleotide polymorphisms (SNPs) in the *TLR4* (rs4986790) and *TLR9* (rs5743836) genes and susceptibility to different clinical forms of *L. infantum* leishmaniasis.

Methods: This case-control study genotyped *TLR4* rs4986790 and *TLR*9 rs5743836 in a Tunisian population with 70 patients (50 VL, 20 CL), and 70 controls. PCR-RFLP and BI-PASA methods were used in the*TLR9* and *TLR4* analysis, respectively. To investigate, we used logistic regression (additive/codominant models).

Results: For *TLR4* rs4986790, genotype frequencies were A/A 94.3% and A/G 5.7% in cases vs. A/A 95.7% and A/G 4.3% in controls (no G/G); no significant associations were found (codominant A/G vs. A/A: OR=1.35, 95% CI 0.29-7.09, *p*=0.698). For *TLR9* rs5743836, T/T 30.0% and T/C 70.0% in cases vs. T/T 37.1% and T/C 62.9% in controls (no C/C); no associations were observed (codominant T/C vs. T/T: OR=1.38, 95% CI 0.68-2.81, *p*=0.371).

Conclusion: These *TLR*4 and *TLR*9 polymorphisms are not associated with *L. infantum* infection outcomes in this Tunisian cohort, suggesting other factors like parasite tropism, drive clinical variability.

## Introduction

*Leishmania (L.) infantum* is a protozoan parasite responsible for visceral leishmaniasis (VL), a severe and potentially fatal systemic form of the disease, as well as cutaneous leishmaniasis (CL), which causes localized lesions. It is now admitted that the progression of the infection is linked to the type of parasite-host cell relationship established. However, there are very different balances between species in terms of the rate of parasite multiplication and the host's immune response [1-3]. Given the crucial role of Toll-like receptors (*TLRs*) in the immune system, genetic variations within these genes can have a significant influence on the host's immune response to pathogens, thereby potentially increasing vulnerability to infection. Some single-nucleotide polymorphisms (SNPs) in *TLR* genes have already been studied in depth in relation to their phenotypic effects in parasitic diseases [4-6]. Indeed, the association between genetic polymorphism of *TLRs* and individual susceptibility or resistance to infectious diseases, including leishmaniasis, has been investigated [7-11]. The identification of these SNPs is essential for understanding the mechanisms of leishmaniasis susceptibility and resistance and could contribute to the development of personalized treatments and prevention strategies.

We aimed to investigate the involvement of two functionally relevant SNPs: the *TLR4 *missense mutation rs4986790 (D299G), known to alter inflammatory signaling, and the *TLR9* promoter polymorphism rs5743836 (T-1237C), which can influence receptor expression in susceptibility to CL and VL.

## Materials and methods

Study population

This case-control study examined TLR4 and TLR9 SNPs in 50 VL patients, 20 CL patients, and 70 controls, with samples collected between 2003 and 2023. The samples were sent to our laboratory for diagnostic purposes, and then the DNAs were conserved at -20°C for further analysis (endemic and immunological). The study population included cases from different regions of Tunisia (Tunis, Monastir, Sousse, and Kairouan). VL and CL cases were diagnosed by clinical examination, parasitological, and molecular methods. Also, PCR for *Leishmania* parasite typing was performed on positive cases as described before [12]. Only *L. infantum* cases were included in this study. For the control group, we selected negative cases of CL and VL, confirmed through parasitological and molecular diagnostics. Thus, 50 negative cases of VL (VL control group) and 20 negative cases of CL (CL control group). Ethical Community of Research in Biological Sciences and Health Care, University of Monastir, issued approval CER-SVS/ISBM 015/2025.

Analysis of *TLR* polymorphism

The SNP rs4986790 is located on the TLR 4 gene on the long arm of chromosome 9. The SNP rs4986790 gene was studied by the PCR-RFLP technique as described by Noori et al. [13] after optimization using the Taguchi method [14]. The following PCR conditions were then used: in a final volume of 50 μl, containing 1× PCR buffer, 2.2 mM MgCl2, 375 μM deoxynucleotides, 0.075 pM of each primer, and 0.5U Taq DNA polymerase with 5ng of DNA. The amplification protocol was: initial denaturation at 94°C for 5 minutes, followed by 35 cycles at 95°C for 20 seconds, 55°C for 30 seconds, 72°C for 30 seconds, and a final extension at 72°C for 10 minutes. Ten µl of the PCR products were then visualized by electrophoresis in a 2% agarose gel containing 5% SYBR Safe. Then, ten µl of PCR product were digested by NcoI according to the supplier's instructions. The PCR products obtained after enzymatic digestion were separated by electrophoresis on a 3% agarose gel containing 5% SYBR Safe. The DNA bands were visualized by exposing the gel to UV light.

The SNP rs5743836 is located in the *TLR *9 gene belonging to the long arm of chromosome 3. We analyzed the SNP rs5743836 using Bidirectional PCR Amplification of Specific Alleles (BI-PASA), which is a PCR conducted with four primers: two external primers, P and Q, and two allele- specific internal primers, A and B. Primers P and Q bind at different distances from the variant to differentiate upstream and downstream PASA reactions [15]. The results were analyzed on an agarose gel. Primers A and B are specific to each allele and bind to the 3′ end of the primer. Depending on the genotype, the Bi-PASA method produces two or three overlapping segments. The PQ segment is always produced and serves as a positive control. The PB and AQ segments are present in heterozygous individuals (WT/M), while only the PB segment is produced in wild-type homozygotes (WT/WT) and only the AQ segment is produced in homozygous mutants (M/M).

The primers chosen to amplify the desired region are those described by Carvalho et al. [16]. The PCR reaction conditions were also optimized by the Taguchi method. Then, we selected the following conditions: in a final volume of 50 μl, containing 1× PCR buffer, 1.25 mM MgCl2, 200 μM deoxynucleotides, 0.4 pM of primers P and Q, and 0.05pM of primers M and W, 0.4U of Taq DNA polymerase with 50ng of DNA. The amplification protocol was: initial denaturation at 94°C for 5 minutes, followed by 35 cycles at 95°C for 20 seconds, 55°C for 30 seconds, 72°C for 30 seconds, and a final extension at 72°C for 10 minutes.

Statistical analysis

Statistical analyses were performed using R Project for Statistical Computing (version 4.5.1; R Core Team (2025). R: A language and environment for statistical computing. R Foundation for Statistical Computing, Vienna, Austria).

The allelic and genotypic frequencies were calculated utilizing the gene-counting method on the SNPassoc 2.1.2 R package.

Genetic association analysis was performed using binary logistic regression under additive and codominant genetic models. The controls served as the reference for calculating odds ratios. Suppose 1 is the ancestral allele and 2 is the altered allele. The codominant model compared the heterozygous (1/2) genotype to the ancestral homozygous (1/1) genotype. The additive model assumed a proportional increase in risk with each additional altered allele 2.

Pearson’s chi-squared test was used to assess inter-group significance for categorical variables and *TLR* genotypes. A p-value of less than 0.05 was considered statistically significant.

## Results

**Figure 1:**
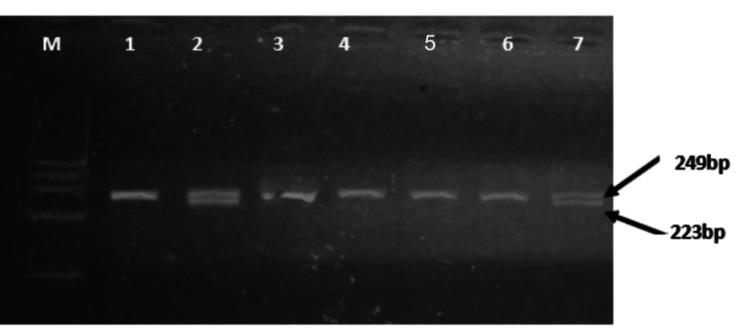
Genotype profiles of SNP rs4986790 in TLR 4 gene 1, 3, 4, 5, and 6: Homozygous genotype samples (A/A) produced a 249 bp single band, 2 and 7: Heterozygous genotype samples (A/G) produced two bands of 249 and 223 bp, M: 100pb ladder size marker.

Analysis of rs4986790 revealed two genotypes (Figure 1). The homozygous A/A genotype produced a single 249 bp band, observed in 133 samples. The heterozygous A/G genotype produced two bands of 249 bp and 223 bp, observed in seven cases. However, we did not observe any cases of the homozygous G/G genotype.

**Figure 2:**
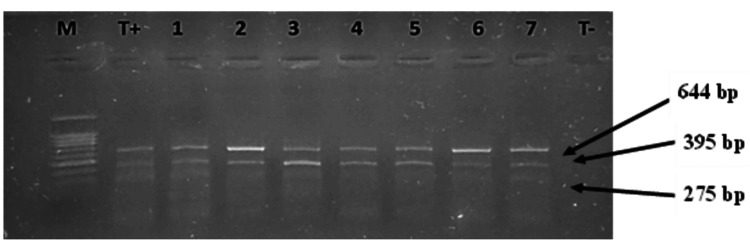
Genotype profiles of SNP rs5743836 in TLR9 gene T+: Positive control, T-: Negative control, 1, 7: Heterozygote genotype samples (C/T) with three bands (644, 395, and 275 bp); 2, 3, 4, 5, 6: Homozygote genotype samples (T/T) with two bands (644 bp and 395 bp). M: 100 bp size marker.

For SNP rs5743836, two different profiles were obtained (Figure 2). The homozygous genotype (T/T), which produces two bands measuring 644 bp and 395 bp, was detected in 47 cases. The heterozygous genotype (T/C) yields three bands (644, 395, and 275 bp), and this genotype was detected in 93 cases. Also, for this SNP, we did not observe any cases of the homozygous C/C genotype.

Allelic and genotypic frequencies of SNPs were then calculated (Table 1). The A/G heterozygous genotype (5%) is less common than the homozygous genotype (95%) for SNP rs4986790. However, for SNP rs5743836, the heterozygous genotype (66.43%) was more frequent than the homozygous genotype (33.57%). However, no significant difference was observed between patients and controls.

**Table 1:** Allelic and genotypic frequencies of analyzed SNPs RA: risk allele.

Genotype and allele distributions: n (%)					
Variant	CL (n = 20)	VL (n = 50)	All Cases (n = 70)	Controls (n = 70)	Total
rs4986790					
A/A	19 (95.0)	47 (94.0)	66 (94.3)	67 (95.7)	133 (95.0%)
A/G	1 (5.0)	3 (6.0)	4 (5.7)	3 (4.3)	7 (5.0)
G/G	0 (0.0)	0 (0.0)	0 (0.0)	0 (0.0)	0 (0.0)
RA (G)	1 (2.5)	3 (3.0)	4 (2.9)	3 (2.1)	7 (5.0)
rs5743836					
T/T	6 (30.0)	15 (30.0)	21 (30.0)	26 (37.1)	47 (33.5)
T/C	14 (70.0)	35 (70.0)	49 (70.0)	44 (62.9)	93 (66.5)
C/C	0 (0.0)	0 (0.0)	0 (0.0)	0 (0.0)	0 (0.0)
RA (C)	14 (35.0)	35 (35.0)	49 (35.0)	44 (31.4)	93 (66.5)

The association between SNPs and leishmaniasis (CL and VL) was evaluated by calculating p- values and ORs (Table 2). No association was detected with p-values > 0.05.

**Table 2:** Association test for leishmaniasis with rs4986790 and rs5743836 polymorphism VL+ : positive cases of VL, VL-: negative cases of VL, CL+: positive cases of CL; CL-: negative cases of CL. †: using Pearson’s chi-squared test

Model of inheritance	All Cases vs. Controls		CL+ vs. CL-	VL+ vs. VL-
rs4986790				
Additive: (G vs. A)	*P*-value†	0.699	1.00	0.648
	OR (95% CI)	1.35 (0.29 - 7.09)	1.00 (0.04 - 26.54)	1.53 (0.24 - 12.03)
Codominant: (A/G vs. A/A)	*P*-value†	0.698	1.00	0.648
	OR (95% CI)	1.35 (0.29 - 7.09)	1.00 (0.04 - 26.54)	1.53 (0.24 - 12.03)
rs5743836				
Additive: (C vs. T)	*P*-value†	0.372	0.736	0.399
	OR (95% CI)	1.38 (0.68 - 2.81)	1.26 (0.33 - 4.87)	1.43 (0.62 - 3.32)
Codominant: (T/C vs. T/T)	*P*-value†	0.371	0.736	0.399
	OR (95% CI)	1.38 (0.68 - 2.81)	1.26 (0.33 - 4.87)	1.43 (0.62 - 3.32)

## Discussion

Genetic and immunological characteristics of the host may play a key role in the pathogenesis of leishmaniasis. Knowledge of the genes that control severe pathology in humans could therefore lead to the development of new, specifically targeted therapeutic approaches. TLR4 gene polymorphisms play a pivotal role in the immune response to various pathogens. This TLR is particularly involved in parasitic infections. Numerous studies have highlighted an association between genetic polymorphism in TLR genes and individual susceptibility or resistance to infectious diseases, including leishmaniasis [4-6]. In this context, we choose to study two SNPs of the TLR4 and TLR9 genes to look for an association with the clinical aspect of *L. infantum leishmaniasis*, which can vary from a simple self-resolving lesion (CL) to a more severe form (VL). We therefore applied two different molecular techniques (RFLP and PCR BI-PASA) to a cohort of 140 samples, including 70 cases of leishmaniasis (50 with VL and 20 with CL) and 70 controls (negative cases). By analyzing the results of the SNP in the TLR4 gene, we found that the A/G heterozygous genotype is present in 5.7% of cases, including 6% (3/50) of VL cases and 5% (1/20) of CL cases. Compared to the control group, we found no significant difference, with 4.5% in VL control cases and 5% in CL control cases. Regarding the second SNP, we unexpectedly found that the SNP of the TLR9 gene is more common than the wild allele in the study population (70%). This may depend on the biological and evolutionary context. The five factors to consider to better understand why certain SNPs may be more common than the wild-type allele would be: mutation, gene flow, non-random mating, genetic drift, and selection [17]. Considering the results of the analysis, we detected the TC heterozygous genotype in 70% of patients, with the same frequency in patients with VL and CL. In comparison with the control group, we observed this genotype in 63.5% of VL controls (31/50) and in 65% of CL controls (13/20). We found no significant association between theTLR4 polymorphism and leishmaniasis development.

Our results corroborate with those of the Iranian team working on 122 patients with VL [18], and the Venezuelan team who studied cases of CL [7]. These teams found no association between TLR 4 polymorphism and leishmaniasis. However, Ejghal et al. provide insights into the possible role of TLR2 and TLR4 variations in VL susceptibility [8]. Also, Ajdary et al. suggested that TLR4 polymorphism could lead to increased susceptibility to the severity of CL caused by *L. major* [9]. Furthermore, it was reported that TLRs (4 and 9) gene polymorphisms can be considered a contributing factor to susceptibility to VL in India [10].

Although no association was observed between the two SNPs and leishmaniasis, the genetic involvement of *TLR*s in the pathology of leishmaniasis is still possible. Indeed, the in vitro study, conducted on mice, suggested the involvement of several loci in the progression of leishmaniasis and showed that the response to Leishmania species is multi-genic [11,19].

Furthermore, the origin of clinical variations in *L. infantum* leishmaniasis could be more likely linked to a balance between both parameters (parasite phenotypic and host immunity system). The dermotropic and viscerotropic tropism of *L. infantum* species could be the major factor of clinical variation. Strains not producing the expected clinical form could be attributed to a specific immune status of the patient. This analysis is supported by the results of the population structure analysis of *L. infantum* strains [20].

This study has several limitations. First, the sample size, particularly for the CL group (n=20), was relatively small, which may have limited our statistical power to detect associations with small effect sizes. Second, the samples were collected over a 20-year period, which could introduce unmeasured cohort effects. Finally, our analysis was restricted to two SNPs; a more comprehensive investigation of the *TLR* pathway or a genome-wide approach would be necessary to fully exclude the role of host genetics.

## Conclusions

In conclusion, this study found no significant association between two SNPs (rs4986790 and rs5743836) and leishmaniasis due to *L. infantum* infection in the Tunisian population. Our findings suggest these specific host genetic factors are not major determinants of disease outcome. Instead, clinical variability is more likely driven by parasite-specific factors, such as strain tropism, with host immune status playing a secondary role.
